# T‐cell engagers in autoimmune rheumatic diseases: From concept to clinical practice

**DOI:** 10.1002/ctm2.70855

**Published:** 2026-10-27

**Authors:** Christina Düsing, Andrea‐Hermina Györfi, Jörg H. W. Distler

**Affiliations:** ^1^ Department of Rheumatology University Hospital Düsseldorf, Medical Faculty of Heinrich‐Heine University Düsseldorf Germany; ^2^ Department of Rheumatology Hiller Research Centre University Hospital Düsseldorf, Medical Faculty of Heinrich‐Heine University Düsseldorf Germany

1

B‐cell depletion has transformed the therapeutic landscape of rheumatic autoimmune diseases and has firmly established B cells as key drivers of immune‐mediated pathology. Since the introduction of anti‐CD20 therapy, substantial advances have been made in understanding the role of B cells in disease pathogenesis and in exploiting their therapeutic potential. However, accumulating evidence indicates that conventional B‐cell depletion is often incomplete, with significant amounts of residual B‐cell populations persisting in lymphoid tissues and inflamed organs, potentially contributing to refractory diseases and relapse.^1^, ^2^ These observations have led to the concept of deep B‐cell depletion, which aims to achieve more complete and sustained elimination of pathogenic B‐cell compartments. This strategy is currently being explored using CAR T‐cell or T‐cell engager (TCE) therapies that target B‐cell antigens such as CD19, CD20 or BCMA. These approaches have shown encouraging results across several severe autoimmune diseases in first case series and small monocentric clinical trials.^3^, ^4^, ^5^, ^6^, ^7^ Beyond improved clinical efficacy, deep B‐cell depletion offers important insights into disease mechanisms and raises the possibility of inducing immune reset^3^, ^4^ leading to durable clinical remission.

Autologous CAR T‐cell therapy, however, requires leukapheresis, individualised manufacturing, lymphodepleting chemotherapy and specialised infrastructure.^3^ TCEs offer an off‐the‐shelf alternative by simultaneously binding CD3 on endogenous T cells and lineage‐specific antigens on B cells or plasma cells, thereby inducing target‐cell cytotoxicity.^8^ Their standardised availability and short pharmacological persistence permit dose adaptation or interruption,^5^ but loss of protective humoral immunity with increased risk of infection^5^, ^6^, ^7^ and systemic T‐cell activation causing cytokine release syndrome (CRS)^5^, ^6^, ^7^ remain significant concerns.

Initial evidence for the efficacy of TCEs in rheumatology came from six patients with multidrug‐refractory rheumatoid arthritis treated with low‐dose CD19×CD3 TCE blinatumomab.^4^ B‐cell depletion was accompanied by clinical improvement, regression of synovitis, reduction of autoantibodies and enrichment of naïve B‐cells in less than three weeks after initiation of blinatumomab.^4^ Subsequent case reports described blinatumomab in severe SSc^9^ and BCMA×CD3 TCE teclistamab in refractory systemic lupus erythematosus (SLE)^7^ and other autoimmune diseases.^6^ These studies established feasibility but did not assess longer‐term outcomes or potential need for a maintenance therapy following TCE‐induced deep B‐cell depletion.

We reported a case series of five patients with anti‐Jo‐1‐positive antisynthetase syndrome (ASyS) treated with blinatumomab and five patients with diffuse cutaneous SSc treated with teclistamab.^5^ All patients had severe and progressive disease and previously failed to respond to multiple conventional immunosuppressants and biologic disease‐modifying anti‐rheumatic drugs (bDMARDs), including, in most cases, rituximab (RTX).^5^ Importantly, all concomitant immunosuppressive and antifibrotic therapies were discontinued before initiation of TCE therapy, allowing the observed effects to be attributed to the intervention itself.^5^ Clinical responses were rapid and profound. In patients with ASyS, blinatumomab induced marked improvement of muscle strength, as evaluated by Manual Muscle Testing‐8, 6‐min walking distance and myositis on magnetic resonance imaging, as well as normalisation of muscle enzymes.^5^ In patients with ASyS‐associated interstitial lung disease (ASyS‐ILD), blinatumomab led to stabilisation of ILD, as evaluated by high‐resolution computed tomography scans and pulmonary function testing.^5^ Follow‐up skeletal muscle biopsies showed regression of inflammatory infiltrates, muscle‐fibre necrosis, complement deposition and major histocompatibility complex class I expression of muscle fibres. Importantly, peripheral B cell depletion was accompanied by elimination of target B cell populations within affected muscle tissue.^5^ In SSc, teclistamab induced broad improvements across multiple disease domains. Skin fibrosis regressed substantially, reflected by clinically meaningful reductions in modified Rodnan skin score (mRSS), while tendon friction rubs and other inflammatory manifestations resolved in all affected patients.^5^ ILD stabilised in all SSc patients.^5^ Overall disease activity improved according to the European Scleroderma Trials and Research Activity Index (EUSTAR‐AI).^5^ Follow‐up skin biopsies demonstrated depletion of plasma cells and B‐lineage cells.^5^

The effects of TCEs on autoantibody responses were heterogeneous. While the BCMA‐targeted TCE teclistamab induced pronounced decreases in autoantibody levels, the effects of the CD19‐targeted TCE blinatumomab were less pronounced.^5^ The titres of the persistent anti‐Jo‐1 antibodies did not parallel clinical activity, and glucocorticoid‐free disease control occurred despite persistent detectable antibodies.^5^ This dissociation suggests that B‐lineage cells contribute through mechanisms beyond autoantibody production, including antigen presentation, cytokine production, T‐cell activation and organisation of inflammatory tissue niches.

A stand‐alone criterion of our study was the use of a maintenance therapy after remission induction with TCEs. TCEs were used to eliminate established pathogenic B‐lineage populations, after which RTX was administered to suppress CD20‐positive precursors.^5^ An ASyS patient who did not receive RTX initially relapsed after B‐cell reconstitution.^5^ This patient responded again to blinatumomab re‐induction and did not demonstrate disease activity during follow‐up under maintenance therapy with RTX.^5^ A recent case series also reported initial responses of SSc patients to blinatumomab, but frequent relapse in the absence of maintenance therapy.^10^ In other patients, RTX maintained disease control during follow‐up, despite previous RTX refractoriness.^5^ RTX may therefore prevent re‐establishment of pathogenic B cell compartments after TCE‐mediated clearance, even when previously ineffective alone.^5^ However, the study cannot establish whether maintenance is universally required, which patients need it, its optimal duration, or whether RTX is the preferred agent or whether other B cell targeting therapies or DMARDs may also be suited.

The comparison between CD19‐ and BCMA‐directed treatment raises the question of target selection. CD19 is expressed across broad stages of B‐cell differentiation, including subsets of plasmablasts, whereas BCMA is enriched on differentiated and long‐lived plasma cells that escape CD20‐ (and to a lesser extent also CD19)‐directed depletion.^5^ However, it is important to note that BCMA‐TCEs also deplete other B cell populations due to minimal expression of BCMA, resulting in broad depletion with complete loss of CD19‐ and CD20‐expressing B cells and plasma cells.^5^ Current evidence does not justify disease‐specific allocation of B cell targets. In our study, treatment target was confounded by diagnosis,^5^ and subsequent reports have described teclistamab responses in anti‐Jo‐1‐positive ASyS^11^ and blinatumomab activity in SSc.^10^ Thus, further studies are required to determine the optimal choices of B cell targets for individual diseases or also for individual patients.

Most patients treated with teclistamab demonstrated loss of autoantibodies on longer‐term follow‐up.^5^ BCMA‐directed TCEs such as teclistamab may therefore induce an immune reset with loss of autoimmunity. However, a claim of a true molecular immune reset requires demonstration of replacement of activated and memory B cells by naïve B cells, with elimination of autoreactive clones and persistence of these changes over time. In the case of such a molecular immune reset, the absence of autoimmunity would allow for sustained treatment‐free remission. So far, longitudinal analyses of immune clonotypes, autoantibody repertoires and tissue niches in patients treated with TCEs have not been published. Further studies should also determine whether TCEs can promote tissue repair or regeneration, as suggested after autologous CD19 CAR T‐cell therapy.^12^

Subsequent studies have extended the efficacy signal. In a SSc series, teclistamab depleted B cells and plasma cells from bone marrow and skin whilst showing clinical improvement.^13^ A series of patients with SSc, immune‐mediated inflammatory myopathies, SLE, undifferentiated connective tissue disease and IgG4‐related disease also reported responses,^14^ although these publications included partially overlapping cohorts.

These encouraging efficacy data shift attention toward the safety profile of this emerging therapeutic strategy. In our cohort, CRS occurred in two of five blinatumomab‐treated patients and all five teclistamab‐treated patients, including grade 3 events.^5^ Six patients developed respiratory infections requiring antibiotics,^5^ and teclistamab caused greater reductions in immunoglobulins and vaccine‐derived antibodies.^5^ In the later series, all patients developed severe hypogammaglobulinemia^14^ and around one‐fourth of patients experienced severe infections.^14^ Two cases with new onset of inflammatory bowel disease‐like colitis^14^ were reported. Moreover, two patients with advanced SSc cardiac involvement died.^13^, ^14^ Although causality was not established, these findings raise concerns about whether advanced cardiac disease may reduce tolerance to TCE‐associated adverse events such as fever, hypotension, fluid shifts, systemic inflammation and infection.

Taken together, our study provides compelling proof‐of‐concept that off‐the‐shelf TCEs can induce profound immune remodelling and meaningful clinical responses in patients with otherwise treatment‐refractory systemic autoimmune rheumatic diseases.^5^ Beyond expanding the therapeutic armamentarium, these findings strengthen the emerging concept that deep depletion of pathogenic B‐cell and plasma‐cell compartments may represent a fundamental principle for achieving durable disease control.

Several important questions, however, remain to be addressed. Longer follow‐up is essential to determine the durability of clinical remission, the kinetics of immune reconstitution, and the long‐term safety of sustained immune depletion. Comparative studies evaluating different TCEs—including agents targeting distinct B‐cell or plasma‐cell antigens—will be required to define the optimal therapeutic approach for individual autoimmune diseases. Likewise, prospective trials are needed to establish the ideal treatment duration and to determine whether maintenance therapy is necessary after induction, and, if so, which strategy is best suited to preserve remission while minimising cumulative immunosuppression.

Further optimisation of safety will be equally important. Future studies should focus on strategies that reduce treatment‐related toxicity without compromising efficacy. These may include optimised step‐up dosing regimens, improved patient selection, biomarker‐guided risk stratification, and prophylactic interventions targeting early inflammatory pathways, such as IL‐6 blockade or other cytokine‐modulating approaches.

Looking ahead, the rapid evolution of immune‐engaging technologies is likely to further expand the therapeutic landscape. Next‐generation multi‐specific constructs, including tri‐specific TCEs^15^ and myeloid engagers, are being designed to enhance target selectivity, improve immune synapse formation and reduce the risk of adverse events. Together, these developments suggest that TCE therapy is unlikely to remain limited to current bispecific molecules but may evolve into a versatile and highly adaptable class of precision immunotherapies capable of reshaping the management of autoimmune disease.

## CONFLICT OF INTEREST STATEMENT

The authors declare no conflict of interest.

## Data Availability

Data sharing is not applicable to this article as no datasets were generated or analysed during the current study.
